# Blastic Plasmacytoid Dendritic Cell Neoplasia: A Single Center Experience

**DOI:** 10.4274/tjh.galenos.2019.2019.0195

**Published:** 2020-02-20

**Authors:** Ahu Senem Demiröz, Cuyan Demirkesen, Ayşe Salihoğlu, Nükhet Tüzüner

**Affiliations:** 1 İstanbul University-Cerrahpasa, Cerrahpasa Faculty of Medicine, Department of Pathology, İstanbul, Turkey; 2 İstanbul University-Cerrahpasa, Cerrahpasa Faculty of Medicine, Department of İnternal Medicine, Hematology, İstanbul, Turkey

**Keywords:** Acute leukemia, Other leukemia, Neoplasia

## Abstract

Blastic plasmacytoid dendritic cell neoplasm (BPDCN) is a rare malignancy with skin tropism. The entity was recently defined and the diagnosis is generally made by skin biopsies. It is necessary to apply appropriate immunohistochemistry to recognize this rare entity. There is no consensus on therapy and the survival rates are low. The aim of this study is to describe the clinical and histopathological features of BPDCN. We retrospectively reviewed 8 BPDCN cases of the Cerrahpaşa Medical Faculty diagnosed between 2005 and 2019. We documented the clinical findings, histopathologic diagnoses, and outcomes. The mean age of the patients was 58.7 years (range=11-86 years), and 7 patients were male. The patients presented with erythematous or purple papules, plaques, and papulonodular or nodular cutaneous lesions. Two had lymphadenomegaly at presentation. In microscopic evaluations, tumor cells infiltrated the entire dermis with a clear-cut subepidermal Grenz zone in all cases. CD4, CD56, and CD123 were the most frequently expressed immunohistochemical markers. The median follow-up of 7 cases was 14 months, ranging from 6 to 48 months. Three patients died of the disease, while 4 patients were still alive. Out of 7 patients, 5 received chemotherapy. We found that the outcomes of some patients were different from others but we did not link any distinct clinical or histopathological characteristics to these different outcomes.

## Abstract (tr)

Blastik plazmasitoid dendritic hücreli neoplazi (BPDCN) nadir görülen, malign bir tümördür. Bu antite son yıllarda tanımlanmış olup, tanı genellikle deri biyopsisinde uygun immunhistokimya panelinin uygulanması ile konulur. Tedavi konusunda tam bir fikir birliği yoktur ve beklenen yaşam süresi kısadır. Bu çalışmanın amacı BPDCN’nin klinik ve histopatolojik özelliklerinin tanımlanmasına katkıda bulunmaktır. Cerrahpaşa Tıp Fakültesi’nde 2005 ile 2014 yılları arası tanı almış olan 8 olgu geriye dönük olarak incelenmiştir. Kliniğe başvuru bulguları, histopatolojik tanı ve klinik gidiş incelenmiştir. Olguların ortalama yaşı 58,7 (11-86) olup, 7 olgu erkektir. Başvuru şikayeti eritematöz ya da mor renkli papül, plak, papülonodül ya da nodüler deri lezyonlarıdır. İki olguda başvuru sırasında lenfadenomegali mevcuttur. Mikroskobik olarak tümör hücreleri subepidermal grenz zon bırakarak tüm dermisi infiltre etmektedir. CD4, CD56 ve CD123 en sık eksprese olan immünhistokimyasal işaretleyicilerdir. Yedi olguda median takip süresi 14 ay (6-48 ay) olup 3 olgu hastalığa bağlı kaybedilmiş, 4 olgu hayattadır. Yedi olgunun 5 tanesi kemoterapi almıştır. Olguların bir kısmında diğerlerine göre farklı klinik gidiş gözlenmiştir, ancak bu durum incelenen klinik ve histokimyasal bulgularla ilişkilendirilememiştir.

## Introduction

Blastic plasmacytoid dendritic cell neoplasm (BPDCN) is a clinically aggressive tumor derived from the precursors of plasmacytoid dendritic cells (PDCs) with a high frequency of cutaneous and bone marrow involvement and leukemic dissemination [1]. The tumor is considered among the “acute myeloid leukemia and related neoplasms” since 2008 [2] and recently it was reclassified as a separate entity in the latest World Health Organization classification scheme. The tumor cells typically express CD4, CD56, CD43, CD45RA, and plasmacytoid dendritic cell antigens (CD123, CD303, TCL1A, CD2AP, SPIB, and type 1 interferon-dependent molecule MX1) [3]. The molecular profile showed that this entity is much more related to myeloid neoplasms [4].

In this study, we present 8 patients with BPDCN from a single center to emphasize the clinical and histopathological features of this rare entity.

## Materials and Methods

Eight cases of BPDCN were retrieved from the archives of the Department of Pathology of the Cerrahpaşa Medical Faculty. Data regarding the clinical features and follow-up were obtained from the patients’ records and their attending physicians. We retrospectively reviewed hematoxylin and eosin and immunohistochemical (IHC) stained slides of skin and bone marrow biopsies. All IHC stainings were performed on a VENTANA BenchMark automated staining system using 4-µm paraffin tissue sections. The primary antibodies used in this study were CD4 (ready to use, Novocastra), CD56 (1:250, Cell Marque), CD123 (1:50, Novocastra), MPO (1:600, DAKO), CD68 (1:400, Novocastra), TdT (1:400, Thermo Scientific), CD7 (1:40, Thermo Scientific), CD20 (1:250, Thermo Scientific), and CD3 (1:300, Novocastra).

## Results

The clinical features of the 8 cases are summarized in Table 1.

The median follow-up of 7 cases was 14 months (ranging from 2 to 48 months). Three patients died of the disease, while 4 were still alive. Of 7 patients, 5 received chemotherapy. Three of them (patients 4, 6, and 7), given the hyper-CVAD regimen, were in remission during the follow-up period. One (patient 5) was given the hyper-CVAD regimen fortified with methotrexate and cytarabine, but he died 8 months after chemotherapy due to systemic involvement. One (the child, patient 8) was given a BFM-ALL high-risk regimen; he was in remission 11 months after the initial diagnosis.

Tumor cells infiltrated the entire dermis with a subepidermal Grenz zone in all cases. In one case, the infiltration reached the subcutaneous fat tissue. The infiltration pattern was diffuse in 5 of the cases, patchy in 1 case, and both diffuse and patchy in 2 cases. Tumor cells were medium-sized with fine chromatin resembling lymphoblasts. Cytoplasm was variably abundant and lacked granules. Mitotic activity was scored as 0-3 in one high-power field (HPF) in 7 cases. A patient with subcutaneous infiltration (14.2%, patient 3) had 2-5 mitotic findings per HPF. Necrosis, vascular invasion, and angiotropism were not detected in any of the cases.

The IHC features are summarized in Table 2.

## Discussion

BPDCN is a recently described entity with an aggressive course. There are only a few series published in the literature. The largest such series comprised 91 patients [5]. This disease is still an obscure entity with many unknowns.

It is typically seen in middle-aged or elderly men, although pediatric cases have also been rarely reported. The age range in our series was 11-86 years with a mean age of 58.7 years. Of 8 patients, one was a child (14.2%). The male:female ratio was 2.5-3:1 [2,6,7,8].

**Figure 1:**
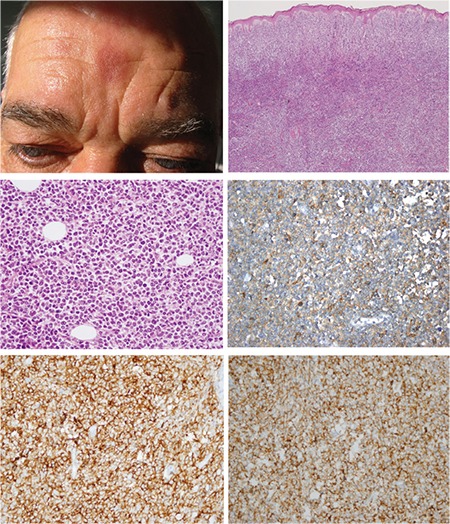
Case 5; A) Papulo-nodular lesion on the patient’s face. B) Diffuse tumoral infiltration including entire dermis and narrow subepidermal grenz zone (hematoxylin & eosin, 400^x^). C) Tumor cell morphology (hematoxylin & eosin, 400^x^). D, E, F) Immunhistochemistry with CD4, CD56, CD123 (400^x^).

**Figure 2:**
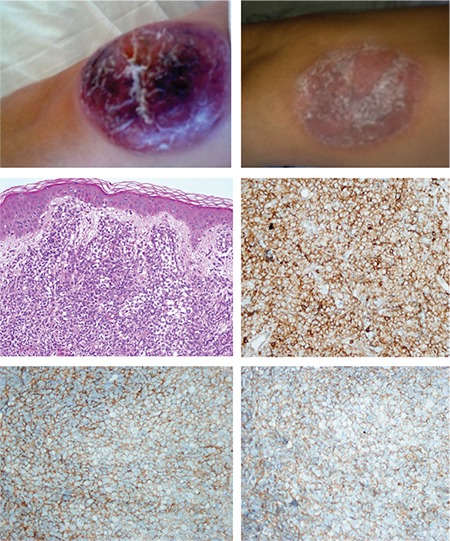
Case 7; A) Endurated nodular lesion on the arm, B) Nodule on the arm was healed after chemotherapy. C) Tumor cell morphology (hematoxylin & eosin, 400^x^). D, E, F) Immunhistochemistry with CD4, CD56, CD123 (400^x^).

The disease tends to involve multiple sites with a predilection for the skin, followed by bone marrow, peripheral blood, and lymph nodes [2]. Approximately 85% of the reported patients presented with cutaneous involvement. There are also a few cases reported without skin lesions [9]. The disease was limited to the skin in half of the cases [10]. The skin lesions may be localized or widespread and the appearance of the lesions varies from small bruise-like areas to violaceous patches, nodules, and ulcerated masses. In our series, 6 patients did not have any extracutaneous involvement; only 2 out of 7 had bone marrow infiltration. The skin lesions in our series consisted of a bruise-like patch in one case (patient 8, the child); macules, plaques, or maculopapular lesions in 3 cases; and nodules in 5 cases with diameters varying from 0.5 to 15 cm. None of them were ulcerated (Figures 1 and 2).

The histology of our series was consistent with the literature [2,11,12]. The only conspicuous feature is that the mitotic rate was higher in the case with extension into the subcutaneous fat (2-5/HPF), and this patient died 4 months after the initial diagnosis. This indicates that as the disease progresses, involvement of the subcutaneous fat tissue takes place [13]. There is only one study showing that patients with high proliferative indexes have significantly better survival [5].

A few tumoral cells in the bone marrow or peripheral blood are likely in the early phases of the disease, as was seen in 2 patients in our series, but overt leukemia is more characteristic for advanced cases or relapses after therapy [13]. Leukemic variants without cutaneous involvement have also been documented [8,12]. Although not verified by biopsy, 2 patients had lymphadenomegaly, which was considered as lymph node involvement (25%). Extracutaneous involvement other than lymph nodes is also seen in the spleen, liver, and tonsils. Cytopenia is the most frequent feature at presentation, whereas B symptoms are not common [3,12].

Circulating normal PDCs and BPDCN both express CD123, TCL1, and CD4. CD56 acquisition by PDCs is associated with oncogenic transformation. PDCs are not present extensively in the skin, but BPDCN expresses CD56 and has skin tropism by binding specifically to E-selectin on dermal endothelial cells and cutaneous T-cells [11,14,15,16]. This may be the cause of the skin predilection of this tumor.

CD4, CD56, and CD123 are the most frequently expressed IHC markers in BPDCN. Neither CD4 nor CD56 negativity excludes the diagnosis [5,11,17,18]. There are no double-negative cases known to date. All of our cases were CD4+ and CD56+. CD123 analysis was performed in 5 cases, which were all positive. It should be kept in mind that CD123 expression is seen also in AML.

Myeloid sarcoma (MS), T-cell lymphoblastic leukemia/lymphoma, NK cell lymphoma/leukemia, and some mature T-cell lymphoma/leukemias are the most frequent morphological mimickers of BPDCN and show some overlapping features [13,19]. The diagnosis depends on exclusion. For the diagnosis of BPDCN, the tumor should be negative for other myelomonocytic, NK, T, and B lineage markers (CD34, CD8, MPO, lysozyme, PAX5, CD20, CD79a, EBV, and T-cell receptor protein). However, the expression of CD33, CD2, CD3, CD7, S100, CD38, and CD10 may be observed [11,12,13]. CD68 immunoreactivity with a paranuclear dot-like pattern is detected in most BPDCN cases, which is similar to our cases, while the staining pattern in MS is diffuse and cytoplasmic [5,10,18,20]. TdT expression is reported in approximately one-third of BPDCN cases, while only one case in our series was positive.

Several IHC markers (TCL-1, BDCA-2, CD2AP) are defined for the differentiation of BPDCN from its mimickers. TCL-1 is expressed in 90% of BPDCN cases and only 17% of c-AML, and it is also seen in a broad variety of B cell lymphoproliferative disorders and some T-cell disorders but is absent in NK cell lineages [19]. BDCA-2 is a specific marker of normal PDCs and is seen in a certain proportion of BPDCN cases [19,21]. CD2AP is a very selective marker for the differentiation of BPDCN from c-AML, but it is also expressed in cortical thymocytes.

Flow cytometry analysis and cytogenetic and clonality studies may also help in diagnosis and in the exclusion of mimickers.

It is pointed out in the literature that the median survival is about 12-14 months [3,11,13]. Advanced age and stage are associated with poor prognostic factors; however, relatively favorable prognosis is seen in children [6]. The only child in our series showed complete remission after treatment. Four of our patients were older than 70 years, and 3 of them died of the disease within 8 months of diagnosis. These outcomes are parallel to the literature and the results are compatible with the expected prognosis of the disease. However, the other 4 patients in our series showed no evidence of disease after the median follow-up of 26.75 months, which is unusual compared to the literature. This may be due to the shorter length of follow-up time. Recently, a few case series with longer overall survival rates were published [22,23,24].

There is no consensus on therapy. Several treatment regimens including therapies for non-Hodgkin lymphoma, ALL, and AML have been used as alternative therapies. Multiagent chemotherapy regimens as in ALL are the most accepted applications for these patients. The disease often relapses after chemotherapy and becomes resistant to the previous drugs [3,4,11,13,23,25].

## Conclusion

In summary, BPDCN is a rare disease with poor prognosis. More studies are necessary to have a better understanding of the disease for proper management.

## Figures and Tables

**Table 1:**
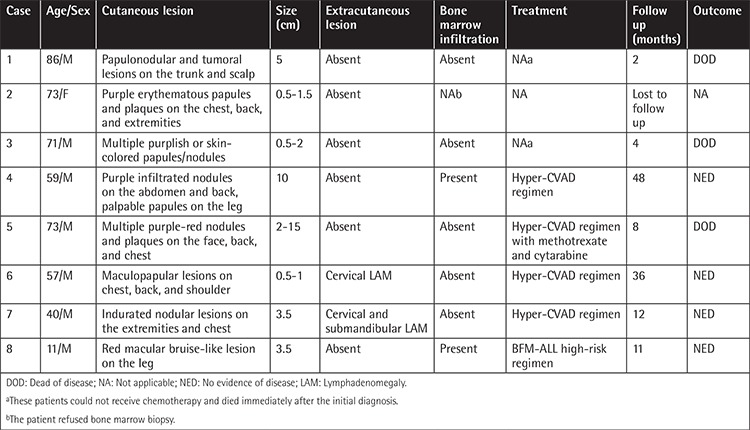
Patient characteristics, clinical data, and outcomes.

**Table 2:**
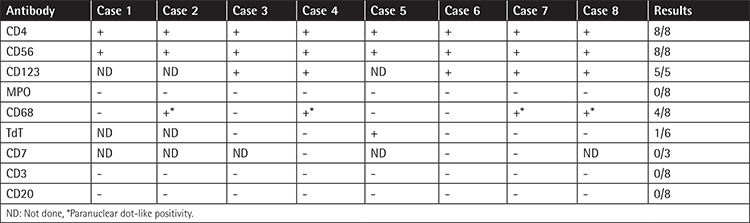
Immunophenotypic profiles.
